# Decursin‐Loaded Nanovesicles Target Macrophages Driven by the Pathological Process of Atherosclerosis

**DOI:** 10.1002/advs.202417489

**Published:** 2025-04-26

**Authors:** Hui Chen, Yifeng Zhang, Mirenuer Aikebaier, Yawei Du, Yan Liu, Qing Zha, Lan Zheng, Shuyao Shan, Yanping Wang, Jiawei Chen, Yiping Li, Ke Yang, Ying Yang, Wenguo Cui

**Affiliations:** ^1^ Department of Endocrinology The Affiliated Hospital of Yunnan University Kunming Yunnan 650021 China; ^2^ Department of Cardiology Shanghai Ninth People's Hospital Shanghai Jiao Tong University School of Medicine Shanghai 200011 China; ^3^ Department of Cardiovascular Medicine Ruijin Hospital Shanghai Jiao Tong University School of Medicine Shanghai 200025 China; ^4^ Department of Orthopaedics Shanghai Key Laboratory for Prevention and Treatment of Bone and Joint Diseases Shanghai Institute of Traumatology and Orthopaedics Ruijin Hospital Shanghai Jiao Tong University School of Medicine Shanghai 200025 China; ^5^ Department of Traditional Chinese Medicine Shanghai Ninth People's Hospital Shanghai Jiao Tong University School of Medicine Shanghai 200011 China

**Keywords:** atherosclerosis, decursin, macrophage, nanovesicles, PKCδ

## Abstract

Atherosclerosis (AS) is a major pathological factor contributing to the mortality associated with ischemic heart disease and is driven primarily by macrophage‐mediated lipid accumulation and inflammatory processes. Conventional cardiovascular pharmacotherapies address these pathological mechanisms but often show limited efficacy, highlighting the need for innovative agents capable of effectively reducing lipid accumulation and inflammation with minimal toxicity. In this study, decursin, a monomer derived from traditional Chinese medicine, is shown to inhibit both lipid accumulation and inflammatory responses in macrophages through direct interaction with protein kinase Cδ (PKCδ), resulting in low cytotoxicity in vitro and negligible toxicity in vivo. To address the short half‐life of decursin, a targeted cascade drug delivery system (ALD@EM), which is specifically designed to target AS pathophysiology, is developed. This system employs ICAM‐1 and VCAM‐1 antibodies for plaque localization and incorporates low‐density lipoproteins (LDLs) to facilitate chemotaxis to lesion sites, with an inner layer of apoptotic endothelial cell membranes to increase macrophage internalization and drug release. As a result, ALD@EM nanovesicles significantly increased the accumulation and therapeutic efficacy of decursin within plaques, substantially reducing lipid deposition and plaque inflammation, thereby offering a novel strategy for targeted AS treatment.

## Graphical abstract

This study presents decursin, a traditional Chinese medicine monomer, that inhibits lipid accumulation and inflammation in atherosclerosis through PKCδ interaction. A novel targeted delivery system (ALD@EM) is developed to overcome decursin's short half‐life, combining antibody‐mediated targeting, LDL‐facilitated chemotaxis, and enhanced macrophage internalization, significantly improving plaque‐targeted therapeutic efficacy.

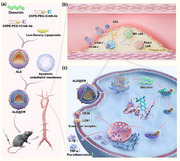

## 1 Introduction

Atherosclerosis (AS) is a chronic inflammatory disease that substantially contributes to major cardiovascular events, including ischemic heart disease and stroke.^[^ ^1^, ^2^ ^]^ The pathophysiology of AS is complex and primarily involves vascular endothelial injury, lipid accumulation, and a self‐perpetuating cycle of inflammatory responses, which together drive the formation and progression of atherosclerotic plaques.^[^ ^3^ ^]^ Dyslipidemia, particularly elevated low‐density lipoprotein (LDL) levels, induces rheological changes in the blood, increasing shear stress on vascular endothelial cells and leading to endothelial dysfunction.^[^ ^4^ ^]^ This dysfunction leads to the upregulation of adhesion molecules such as vascular cell adhesion molecule‐1 (VCAM‐1) and intercellular adhesion molecule‐1 (ICAM‐1),^[^ ^5^ ^]^ facilitating the adhesion of circulating monocytes to the damaged endothelium and their migration into the subendothelial space, where they differentiate into macrophages.^[^ ^6^ ^]^ Under the influence of oxidized LDL (Ox‐LDL), these macrophages internalize Ox‐LDL via scavenger receptors such as CD36 and LOX‐1, transforming into lipid‐laden foam cells.^[^ ^7^, ^8^ ^]^ The subsequent apoptosis and necrosis of foam cells release proinflammatory cytokines, including interleukin‐1β (IL‐1β), further exacerbating local inflammation and creating an inflammatory amplification cascade. This process promotes the formation and destabilization of atherosclerotic plaques, increasing the risk of cardiovascular events.^[^ ^9^, ^10^, ^11^, ^12^ ^]^ Consequently, targeting macrophages to inhibit lipid accumulation and inflammatory responses represents a highly effective strategy for treating atherosclerosis. Clinically, statins are first‐line therapeutic agents that function primarily by inhibiting cholesterol synthesis and lowering plasma LDL‐C levels.^[^ ^13^, ^14^ ^]^ However, large‐scale clinical trials, such as HPS, ASCOT‐LLA, CORONA, and JUPITER, have identified potential risks associated with statin use, including an increased risk of new‐onset diabetes and contraindications for patients with renal insufficiency. Additionally, racial variations in drug tolerance limit their broad applicability.^[^ ^15^, ^16^, ^17^, ^18^ ^]^ Emerging anti‐inflammatory therapies, such as the monoclonal antibody canakinumab, which targets IL‐1β, have shown efficacy in reducing cardiovascular event risk without affecting LDL‐C levels. Nevertheless, the risk of severe infections, including sepsis and pneumonia, limits their clinical use.^[^ ^19^ ^]^ Both lipid‐lowering and anti‐inflammatory treatments can potentially cause systemic toxicities related to their administration routes. Consequently, the development of novel antiatherosclerotic drugs that are both efficacious and safe, particularly those capable of targeted plaque delivery, remains a critical challenge in the cardiovascular field.

**Scheme 1:**
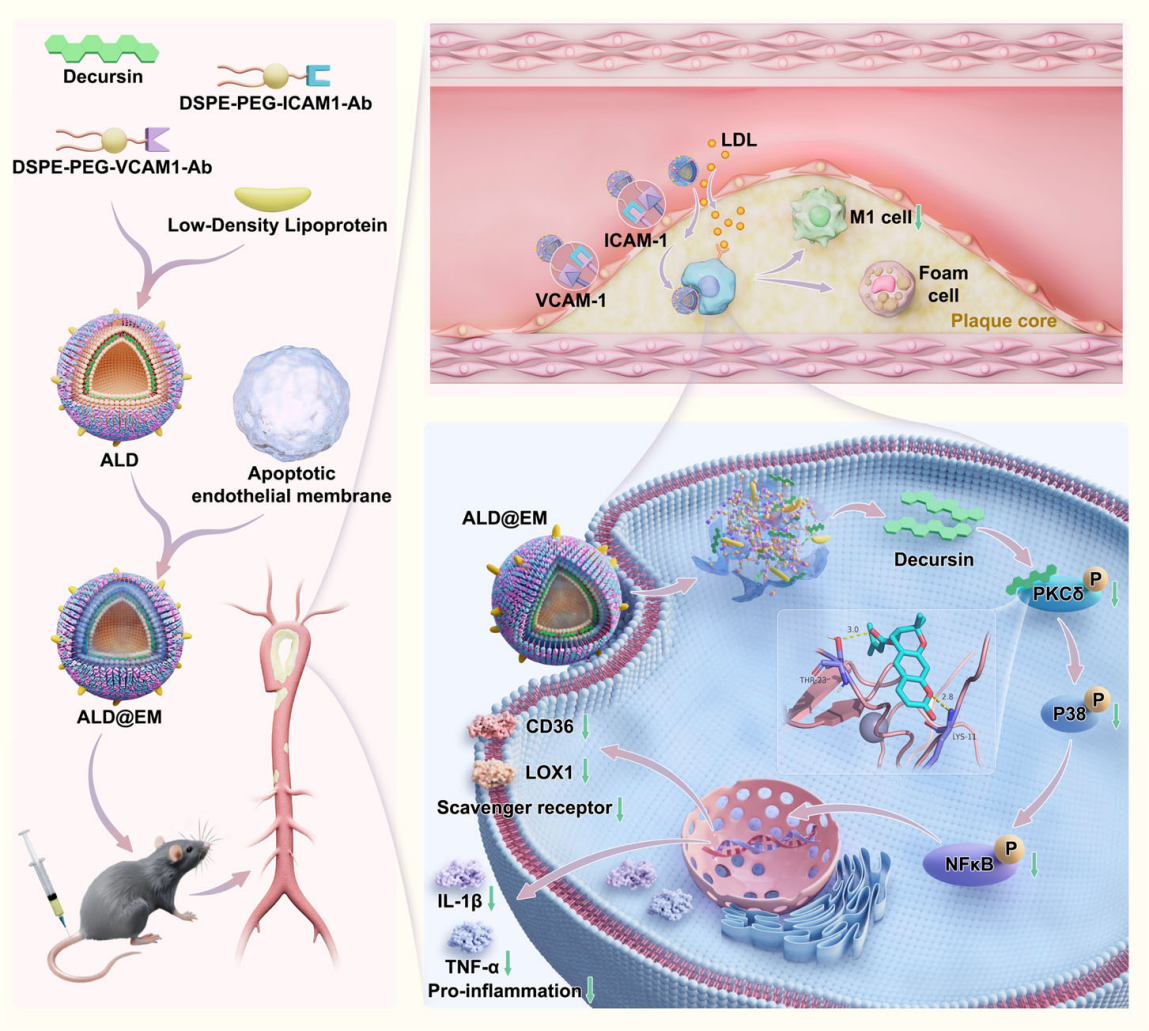
Design and Mechanism of the ALD@EM Macrophage‐Targeted Drug Delivery System. a) Schematic illustration of ALD@EM synthesis. b) Cascade‐targeting mechanism of ALD@EM in macrophages within atherosclerotic plaques. c) The regulatory mechanism of decursin following its internalization into macrophages.

In response to the current challenges in drug development, our research has strategically focused on plant‐derived compounds.^[^ ^20^ ^]^ Plant‐based medications, a cornerstone of traditional Chinese medicine, boast a long‐standing history of clinical use, affordability, and reduced toxicity. These compounds show good potential in treating chronic diseases, particularly cardiovascular conditions. Among them, decursin is extracted from the roots of *Angelica gigas* Nakai of the Umbelliferae family and has garnered considerable attention for its robust multitarget anti‐inflammatory properties.^[^ ^21^, ^22^, ^23^, ^24^ ^]^ Decursin effectively inhibits adipogenesis in adipose stem cells by activating the β‐catenin signaling pathway, underscoring its crucial role in regulating lipid metabolism.^[^ ^25^ ^]^ The ability of this compound to prevent lipid accumulation and reduce inflammation makes it a highly promising antiatherosclerotic agent, aligning seamlessly with current therapeutic strategies aimed at mitigating macrophage lipid uptake and inflammatory responses. Notably, animal studies have confirmed that decursin is nontoxic at doses up to 2000 mg kg^−1^ body weight, highlighting its potential as a safe and efficacious treatment for atherosclerosis.^[^ ^26^ ^]^ Our research team has extensively explored the mechanisms by which decursin inhibits macrophage activation, revealing that it suppresses the expression of the key scavenger receptors CD36 and LOX‐1. This suppression reduces the uptake of Ox‐LDL and diminishes foam cell formation. Additionally, decursin attenuates the secretion of proinflammatory cytokines, thereby alleviating the inflammatory environment within atherosclerotic plaques. These cellular actions are mediated through the direct binding of decursin to protein kinase Cδ (PKCδ), which inhibits its phosphorylation and subsequent activation of downstream signaling pathways, such as p38 and NF‐κB, effectively counteracting both lipid accumulation and inflammatory responses in macrophages (**Scheme** **1**c).

Despite its promising pharmacological properties, several critical limitations have hindered its therapeutic development. First, decursin has poor bioavailability (approximately 3.8% in rodent models) due to extensive first‐pass metabolism and low aqueous solubility.^[^ ^27^ ^]^ Second, its rapid plasma clearance (t₁/₂ = 1.26 h) severely limits sustained therapeutic effects.^[^ ^28^ ^]^ The clinical application of decursin is substantially limited by its short half‐life following intravenous injection, as the compound is rapidly metabolized and degraded before reaching targeted lesion sites.^[^ ^29^ ^]^ To address this challenge, researchers need to develop a precise delivery system capable of transporting decursin directly to the core of atherosclerotic plaques, with a particular focus on targeting macrophages involved in plaque formation. Traditional drug delivery methods, such as polymer or biomembrane encapsulation, often lack the clinical precision required for effective macrophage activation targeting.^[^ ^30^, ^31^ ^]^ To bridge this gap, we have developed an innovative targeted cascade drug delivery system (ALD@EM) based on the pathophysiological mechanisms of atherosclerosis. This system promotes the in vivo stability and targeting capability of decursin, thereby increasing its antiatherosclerotic efficacy and providing a novel strategy for the precise treatment of atherosclerosis. The ALD@EM design incorporates a multilevel targeting approach (Scheme 1a,b): the outer layer of the nanovesicles consists of DSPE‐PEG‐ICAM1 and DSPE‐PEG‐VCAM1 antibodies conjugated via DSPE‐PEG‐NHS, enabling specific plaque targeting. The middle layer includes LDL, which utilizes the impaired endothelial barrier within plaques to diffuse into the intima and accumulate in the plaque core, guiding ALD@EM toward the plaque interior through chemotaxis. The innermost layer is composed of biomembranes derived from apoptotic endothelial cells engineered to activate macrophage efferocytosis within the plaque environment. This phenomenon promotes the recognition, internalization, and degradation of ALD@EM by macrophages, leading to the release of decursin. Once released, decursin effectively reduces lipid accumulation and inflammatory responses in macrophages, providing therapeutic benefits against atherosclerosis. This cascade delivery mechanism ensures the precise accumulation of ALD@EM in plaque macrophages, suggesting a novel and effective strategy for targeted atherosclerosis treatment. This innovative approach not only overcomes the limitations of current therapies but also utilizes the pathophysiological features of plaque formation, promising increased therapeutic efficacy and minimal systemic side effects.

## 2 Results

### 2.1 Therapeutic Efficacy of Decursin In Vivo and Vitro

The potential therapeutic effects of decursin, a traditional Chinese herbal monomer, on the pathological progression of atherosclerosis have not been previously reported in the literature. To explore its antiatherosclerotic properties, we conducted a series of comprehensive in vivo and in vitro experiments. In vivo, apolipoprotein E‐deficient (ApoE‐/‐) mice, an established model for atherosclerosis research, were fed a high‐fat diet (HFD) and divided into two experimental groups (Figure S1a, Supporting Information). Starting from the fourth week after HFD treatment, the mice received weekly intravenous injections of either vehicle or decursin, the latter of which was administered at a standardized dose of 100 µL with a concentration of 5 mg mL^−1^, over a 10‐week period. At the conclusion of the treatment, the aortic samples were subjected to oil red O (ORO) staining, a well‐established method for identifying plaques. The ORO staining results revealed distinct plaque formation in the control group treated with vehicle alone, whereas the decursin‐treated group demonstrated an ≈10% reduction in plaque area compared with the control group (Figure S1b,d, Supporting Information). These findings provide preliminary evidence of an inhibitory effect of decursin on atherosclerotic disease progression, suggesting its potential utility in reducing plaque burden.

In vitro, the effects of decursin on lipid uptake in macrophages, a critical cellular process contributing to foam cell formation and plaque development, were investigated using the RAW264.7 murine macrophage line. The cells were treated with vehicle, Ox‐LDL, or a combination of Ox‐LDL and decursin for 24 h. The intracellular lipid content was measured using Nile red fluorescence staining, followed by confocal microscopy imaging. As anticipated, Ox‐LDL treatment significantly increased lipid uptake in macrophages, resulting in pronounced intracellular lipid accumulation. However, co‐treatment with decursin markedly reduced this lipid load, effectively reversing the Ox‐LDL‐induced changes (Figure S1c,e, Supporting Information). Additionally, consistent with the anti‐inflammatory effects reported for other bioactive compounds, decursin treatment suppressed the expression of key proinflammatory cytokines, including IL‐1β and TNF‐α (Figure S2, Supporting Information). This finding underscores the potential mechanistic basis for the antiatherosclerotic effects of decursin, as inflammation is a critical driver of plaque instability and progression in atherosclerosis. Collectively, the data from these in vivo and in vitro experiments support the hypothesis that decursin plays a protective role in atherosclerotic disease by mitigating both lipid accumulation and inflammatory responses. These findings lay the groundwork for future research into the clinical application of decursin as a promising therapeutic agent for cardiovascular disease prevention and management.

### 2.2 Synthesis and Characterization of ALD@EM

**Figure 1:**
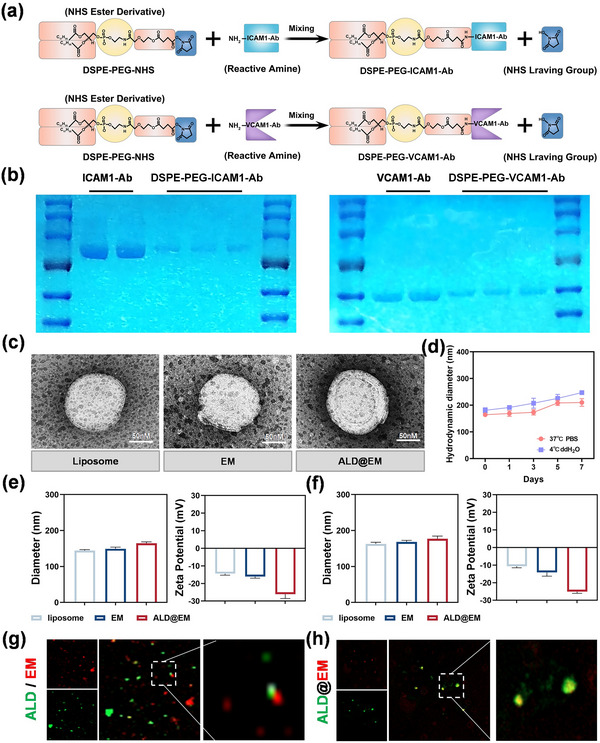
Synthesis and Characterization of ALD@EM. a) Chemical synthesis of DSPE‐PEG‐ICAM1‐Ab and DSPE‐PEG‐VCAM1‐Ab. b) Coomassie blue staining via SDS‒PAGE was employed to semiquantitatively assess the conjugation of antibodies (ICAM1‐Ab and VCAM1‐Ab) to DSPE‐PEG‐NHS. c) Representative TEM images of EM and ALD@EM. d) Hydrodynamic diameter of ALD@EM. e,f) Diameter and zeta potential measurements of EM and ALD@EM following conventional storage (e) and 24‐h room temperature storage (f). (*n* = 5). g,h) CLSM images of EM and ALD in the mixed system (EM + ALD) or EM/ALD, labeled with Dil (red) and FITC (green), respectively.

DSPE‐PEG‐NHS is an ideal bifunctional linker for our application because of its unique amphipathic structure: the hydrophobic DSPE portion readily integrates into lipid membranes, whereas the hydrophilic PEG chain extends outward with a terminal NHS ester for facile bioconjugation. Compared with conventional conjugation methods, this phospholipid‐PEG conjugate has demonstrated superior performance in terms of antibody modification, offering increased stability, reduced immunogenicity, and improved pharmacokinetic profiles. We optimized the conjugation reaction between DSPE‐PEG‐NHS and our targeting antibodies (ICAM1‐Ab and VCAM1‐Ab) through careful control of the reaction parameters (pH 7.4, 4 °C, 24 h incubation), achieving conjugation efficiencies of ≈85% and 82%, respectively, as determined by HPLC analysis. The resulting DSPE‐PEG‐ICAM1‐Ab and DSPE‐PEG‐VCAM1‐Ab conjugates (**Figure** **1**a) retained their immunoreactivity while gaining the lipid‐anchoring capabilities essential for stable membrane incorporation.

To further confirm the successful modification of ALD@EM with ICAM1‐Ab and VCAM1‐Ab, we employed sodium dodecyl sulfate‒polyacrylamide gel electrophoresis (SDS‒PAGE) to detect the incorporation of these components (Figure 1b). As shown in Figure 1c, transmission electron microscopy (TEM) images of ALD@EM reveal a unique core–shell structure characterized by a nanosized inner core enveloped by a membrane shell. With the EM coating, ALD demonstrated much greater stability. As shown in Figure 1d, EM/ALD maintained a certain particle size distribution range in phosphate‐buffered saline (PBS) and cell culture media at 37 °C and showed <15% particle size variation within 5 days, indicating good colloidal stability in physiological environments. Dynamic light scattering (DLS) measurements indicate that the size of ALD@EM is ≈164 nm, which is slightly larger than that of EM, with a zeta potential of ≈−27 mV (Figure 1e). Importantly, both the particle size and zeta potential remained statistically unchanged (p > 0.05) after storage at room temperature (25 °C) for 24 h, with <5% variation in all the measured parameters (Figure 1f). This remarkable stability under physiologically relevant conditions suggests strong potential for in vivo applications without premature degradation or cargo leakage.

Moreover, confocal laser scanning microscopy (CLSM) analysis (Figure 1g,h) revealed a high overlap of ALD and EM fluorescence signals, which were labeled with FITC and Dil dyes, respectively. In contrast, the non extruded ALD and EM mixtures presented distinct red and green fluorescence signals, indicating their separation. These findings provide further confirmation of the successful coating of the apoptotic membrane onto ALD. The encapsulation efficiency of decursin in ALD@EM was 83.7 ± 4.2%, and the drug loading capacity was 2.31 ± 0.18% (w/w), as determined via HPLC quantification of the free drug in the supernatant after ultracentrifugation.

### 2.3 Safety Evaluation In Vivo and In Vitro

**Figure 2:**
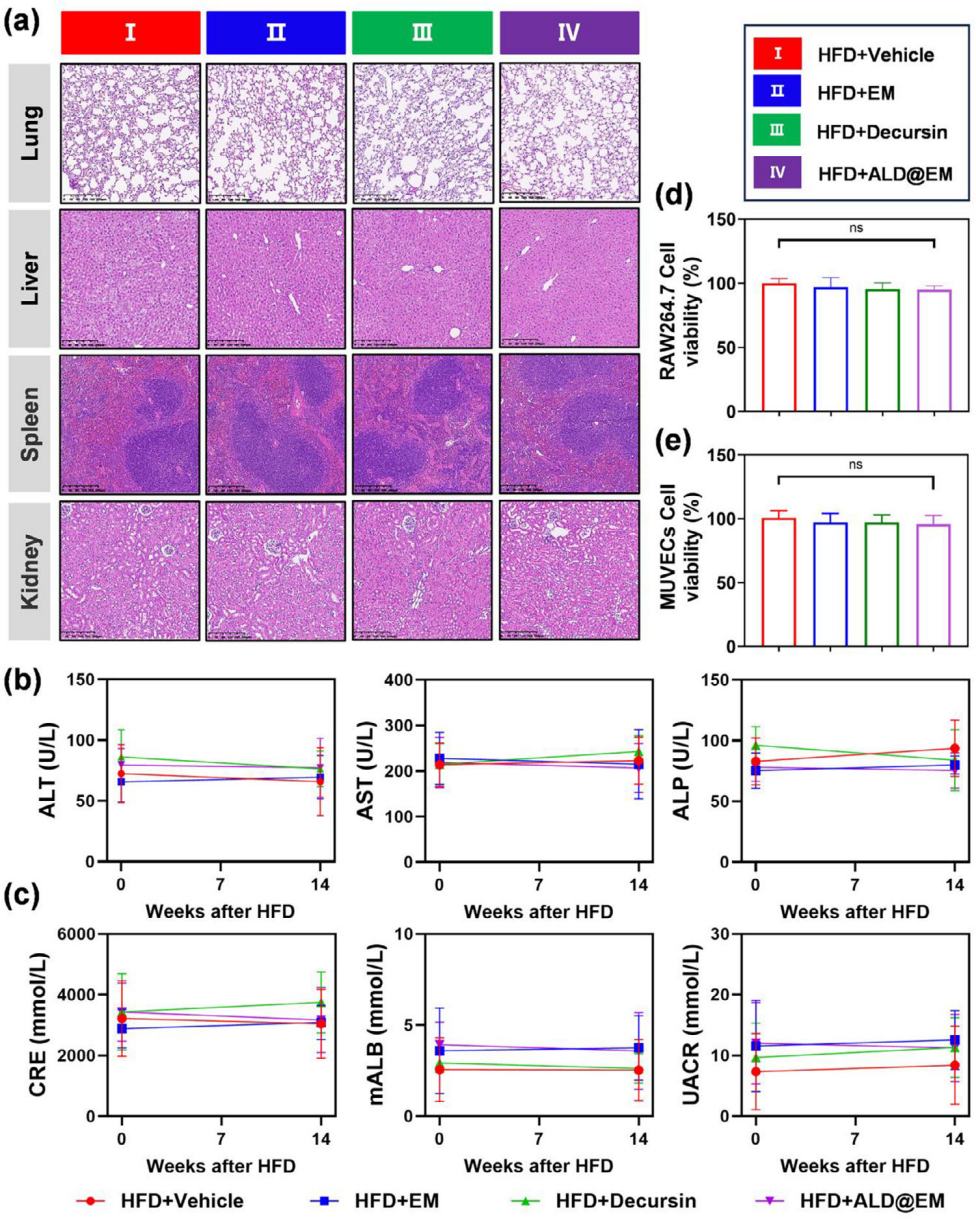
Biocompatibility evaluation of ALD@EM in vitro and in vivo. a) H&E staining of noncardiac organs (lung, spleen, liver, and kidney) at 14 weeks after HFD feeding. b,c) Assessment of key liver function indicators (ALT, AST, and ALP) (b) and renal function indicators (CRE, mALB, and UACR) (c) at 14 weeks after HFD feeding (*n* = 5). d,e) Proliferation of RAW264.7 (d) and MUVECs (e) cocultured with ALD@EM as assessed by a CCK‐8 assay on Day 3 (*n* = 5). OD values were measured at 450 nm. The data are expressed as the means ± s.d.s. NS indicates not significant.

Atherosclerosis, a chronic condition requiring sustained pharmacological intervention, underscores the critical importance of the safety profile of therapeutic agents. To thoroughly assess the systemic toxicity of vehicle, EM, decursin, and ALD@EM, we performed hematoxylin and eosin (H&E) staining on major noncardiac organs, including the lungs, kidneys, liver, and spleen, in each mouse group prior to in vivo safety evaluations. At 14 weeks after HFD induction, these organs, along with the aorta, were harvested for analysis. H&E staining revealed no significant pathological differences among the treatment groups (**Figure** **2**a), indicating the absence of overt tissue damage. Furthermore, urine and blood samples collected at the 14‐week mark were analyzed for key renal function indicators (CRE, mALB, and UACR) and liver function markers (ALT, AST, and ALP). The results revealed no significant changes in hepatic or renal function parameters between the ALD@EM injection group and the vehicle control group (Figure 2b,c), confirming the high biocompatibility of ALD@EM. In support of the in vivo findings, in vitro cytotoxicity assays conducted on RAW264.7 cells and MUVECs treated with vehicle, EM, decursin, or ALD@EM revealed no statistically significant differences in cell proliferation after 24 h of coincubation, as demonstrated by CCK‐8 assays (Figure 2d,e). Collectively, these findings suggest that EM, decursin, and ALD@EM exhibit favorable biocompatibility profiles both in vivo and in vitro, supporting their potential for chronic administration in the treatment of atherosclerosis.

### 2.4 Evaluation of the In Vivo‐ and In Vitro‐Targeted Delivery Efficiency

**Figure 3:**
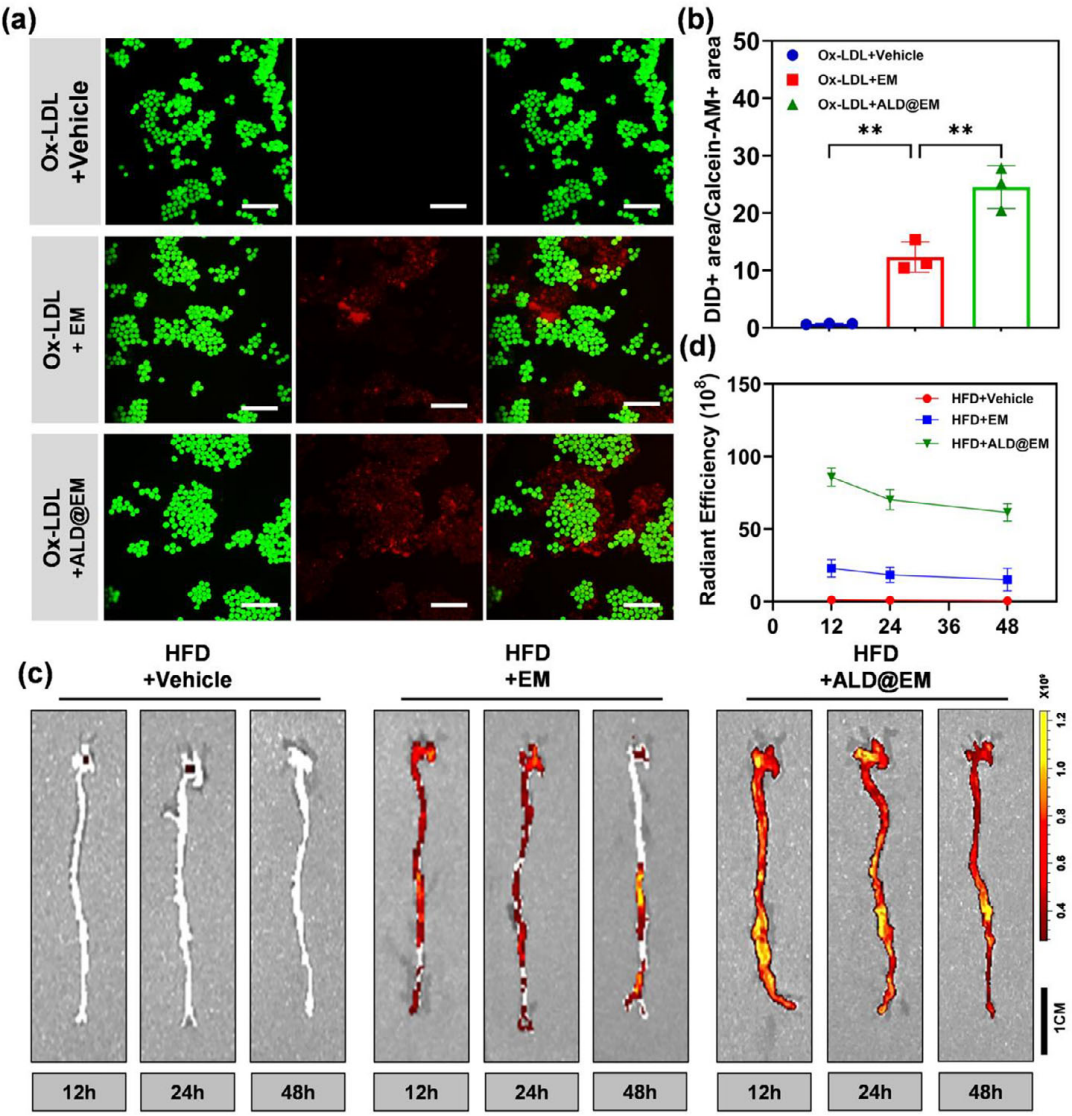
Evaluation of the efficacy of targeted delivery in vivo and in vitro. a) Microscope images showing the binding of DiD‐labeled EM and ALD@EM to RAW264.7 cells after incubation with Ox‐LDL. Scale bars, 100 µm. b) Quantitation of DiD‐labeled EM and ALD@EM remaining bound to RAW264.7 cells after elution (*n* = 3). c,d) Fluorescence imaging (c) and radiant efficiency quantification (d) in mice at 12 h, 24 h, and 48 h after intravenous injection of DiD‐labeled EM or ALD@EM (*n* = 5). The data are expressed as the means ± s.d.s. NS indicates not significant. ^**^ *p* < 0.01.

The targeting capabilities of ALD@EM in atherosclerotic environments were systematically evaluated through comprehensive in vitro and in vivo analyses. In vitro, a macrophage‐level atherosclerosis model was established by stimulating RAW264.7 cells with Ox‐LDL. Both EM and ALD@EM were fluorescently labeled with DiIC18(5) dye (DiD) to assess their targeting effects on macrophages within atherosclerotic plaques. Fluorescence microscopy demonstrated that after coincubation and subsequent PBS washing to remove unbound particles, ALD@EM exhibited significantly stronger binding to damaged RAW264.7 cells than EM (**Figure** **3**a,b). These findings indicate that ALD@EM possesses increased specific targeting and retention capabilities of macrophages affected by atherosclerotic injury.

For the in vivo evaluation of targeting specificity, atherosclerotic model mice were intravenously injected with DiIC18(7)‐labeled EM or ALD@EM. In vivo imaging revealed significantly greater fluorescence in the cardiac region in both nanovesicle groups than in the control group, with the ALD@EM NP group exhibiting the highest fluorescence intensity and prolonged signal duration (Figure 3c,d). Notably, substantial fluorescence persisted within the atherosclerotic plaques even 48 h post‐injection, indicating sustained accumulation. These findings collectively demonstrate that ALD@EM possesses superior targeting specificity and prolonged retention in atherosclerotic plaque tissues compared with EM alone. This optimal targeting and accumulation are crucial for increasing the efficacy and duration of drug action in vivo, potentially enabling more effective therapeutic strategies against atherosclerosis.

### 2.5 Therapeutic Efficacy of ALD@EM In Vivo and In Vitro

**Figure 4:**
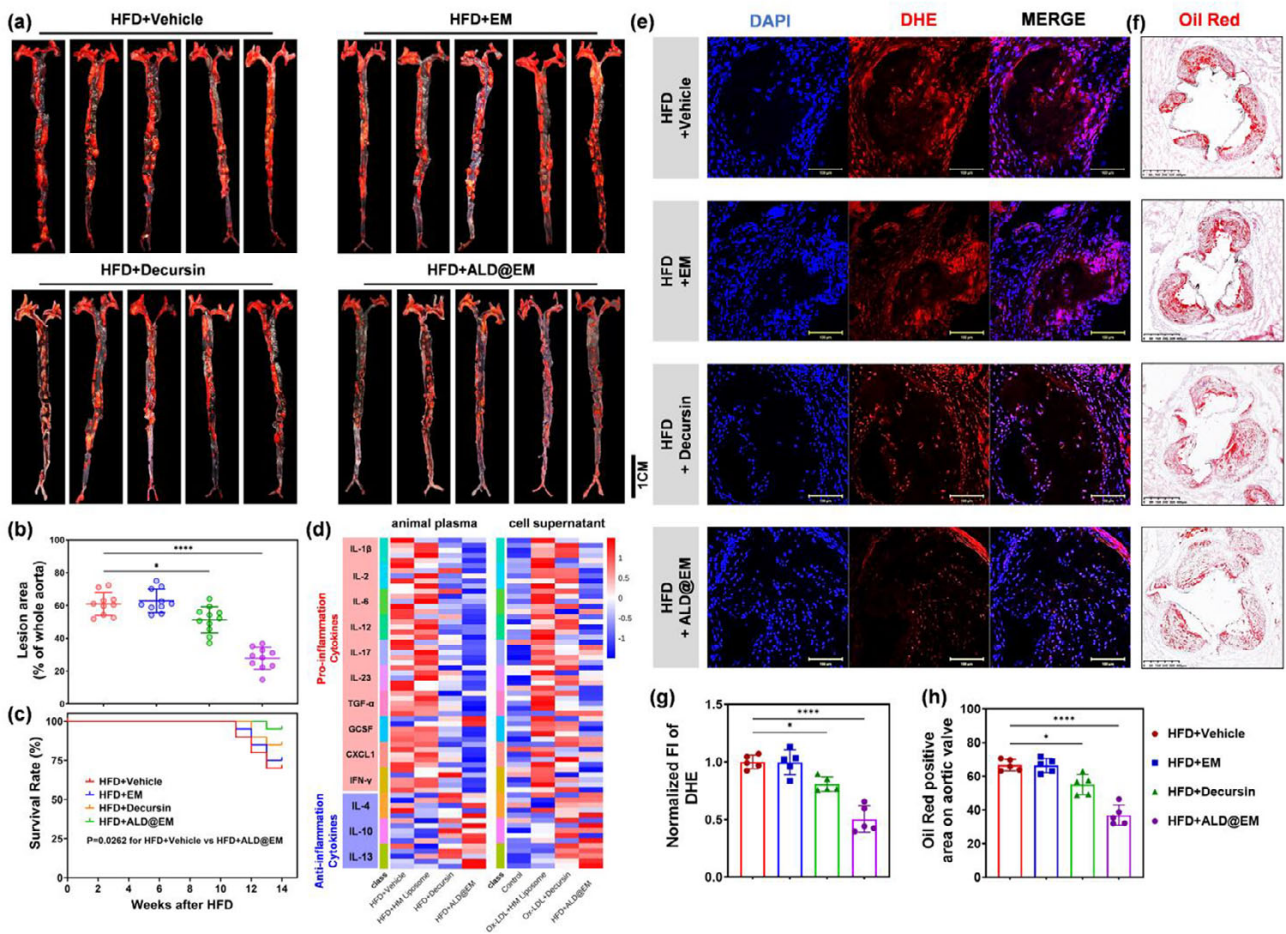
Effects of ALD@EM on Atherosclerosis Progression. a) Representative images of aortas stained with ORO after various treatments at 14 weeks after HFD. b) Quantification of the ORO‐positive lesion area relative to the whole aorta (*n* = 10). c) Survival curves of mice fed an HFD for 14 weeks (*n* = 20). d) Quantification of inflammatory cytokines in mouse plasma and RAW264.7 cell supernatants (*n* = 5). e,f) Representative images of aortic valves stained with DHE fluorescent stain (e) and ORO (f). g) Quantification of the normalized fluorescence intensity (FI) of DHE‐stained aortic valves (*n* = 5). h) Percentage of ORO‐positive area in aortic valves (*n* = 5). The data are expressed as the means ± s.d.s. NS indicates not significant. ^*^ *p* < 0.05 and ^****^ *p* < 0.0001.

To assess the efficacy of ALD@EM in increasing the antiatherosclerotic effects of decursin, we divided ApoE‐/‐mice into four groups and fed them an HFD. Starting at week 4, each group received weekly intravenous injections of 100 µL of vehicle, EM, decursin, or ALD@EM for a total of 10 weeks, as shown in Figure S3, Supporting Information. Analysis of the aortas using ORO staining to detect atherosclerotic plaques revealed that the ALD@EM NP treatment group exhibited a significant 45.6% reduction in plaque area compared with the control group (**Figure** **4**a,b). This change represents a ≈fourfold improvement over decursin alone, which achieved only a 10% reduction, demonstrating that ALD@EM substantially promotes the therapeutic effect of decursin by targeting macrophages within the plaque core.

Survival curve analysis revealed that decursin reduced mortality in atherosclerotic mice, with the ALD@EM group showing the highest survival rate and lowest mortality among all groups (Figure 4c). Additionally, measurements of inflammatory cytokine levels in both the serum samples and the Ox‐LDL‐stimulated RAW264.7 cell cultures revealed decreased proinflammatory cytokine levels and increased anti‐inflammatory cytokine levels in the decursin and ALD@EM groups (Figure 4d), indicating anti‐inflammatory effect. Lipid profiling at 14 weeks after HFD consumption revealed that ALD@EM reduced total cholesterol (T‐CHO), triglyceride (TG), and LDL‐c levels while increasing high‐density lipoprotein cholesterol (HDL‐c) levels (Figure S4, Supporting Information). Assessment of ROS production in aortic valve tissues using dihydroethidium (DHE) immunofluorescence staining revealed that, compared with the control, decursin reduced ROS levels, and ALD@EM significantly promoted this effect (Figure 4e,g). Furthermore, ORO staining of the aortic valve region revealed that, compared with the control, decursin decreased lipid accumulation and plaque formation, and this therapeutic effect was further amplified by ALD@EM NP treatment (Figure 4f,h). Collectively, these findings suggest that ALD@EM promotes the antiatherosclerotic effects of decursin through improved targeting of macrophages, leading to significant reductions in plaque formation, inflammation, lipid levels, and oxidative stress.

### 2.6 ALD@EM Significantly Improves Cardiac Function

**Figure 5:**
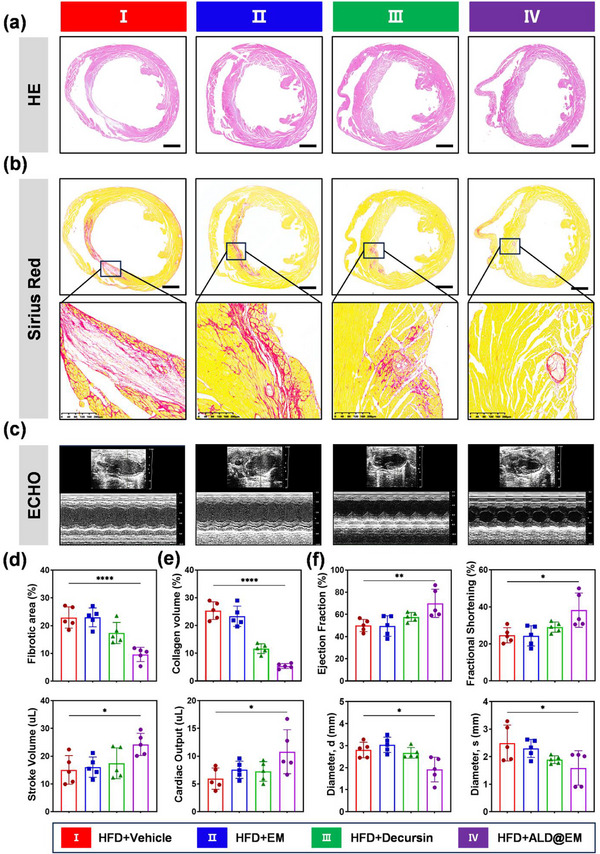
ALD@EM Significantly Improves Cardiac Function. a,b) Representative images of H&E‐stained (a) and Sirius Red‐stained (b) mouse cardiac sections at 14 weeks after HFD feeding. c) Representative M‐mode echocardiographic images at 14 weeks after HFD feeding. d) Quantitative analysis of fibrotic tissue via MT staining at 14 weeks after HFD consumption (*n* = 5). e) Collagen volume was analyzed in Sirius Red‐stained cardiac sections (*n* = 5). f) Ejection fraction, fractional shortening, stroke volume, cardiac output, diameter,d, and diameter,s were assessed via M‐mode echocardiography (*n* = 5). The data are expressed as the means ± s.d.s. NS indicates not significant. ^*^ *p* < 0.05, ^**^ *p* < 0.01, and ^****^ *p* < 0.0001.

Atherosclerosis is a progressive inflammatory disorder characterized by arterial plaque formation that can ultimately lead to coronary artery occlusion, myocardial ischemia, and infarction. These pathological events trigger a cascade of deleterious processes, including cardiomyocyte death, replacement fibrosis, ventricular remodeling, and eventual cardiac dysfunction. To evaluate whether our targeted drug delivery system could mitigate these adverse cardiac manifestations, we conducted comprehensive histopathological analyses of myocardial tissue samples across multiple experimental groups. Hematoxylin and eosin (H&E) staining revealed striking differences in the myocardial architecture between the treatment groups (**Figure** **5**a). The control animals presented extensive regions of cardiomyocyte loss characterized by cellular fragmentation, nuclear pyknosis, and disrupted myofibrillar arrangement. In contrast, the ALD@EM‐treated subjects demonstrated remarkably preserved myocardial integrity with significantly reduced focal necrosis and maintained intercalated disc structures (Figure 5d). To specifically assess collagen deposition and fibrotic remodeling, we employed Sirius Red staining, which provides superior specificity for collagen fibers through binding to basic amino groups in collagen molecules (Figure 5b). This aberrant extracellular matrix deposition was particularly pronounced in the peri‐infarct border zone and extended substantially into remote myocardial regions, suggesting adverse ventricular remodeling. Conversely, the ALD@EM‐treated samples presented markedly reduced collagen content (Figure 5e).

Cardiac function was further assessed using echocardiography. The results demonstrated that both decursin and ALD@EM markedly improved cardiac function parameters, such as EF, FS, SV, CO, LVDd, and LVSd, in atherosclerotic mice. Notably, the improvements were more pronounced with ALD@EM treatment (Figure 5c,f), indicating superior efficacy in promoting cardiac performance. In summary, these findings strongly indicate that ALD@EM can mitigate myocardial infarction caused by terminal artery plaque occlusion by slowing the progression of atherosclerosis and improving cardiac function. The significant reduction in myocardial injury markers and increase in cardiac function parameters highlight the potential of ALD@EM as a therapeutic agent for atherosclerosis‐induced cardiac dysfunction.

### 2.7 Decursin Interaction with PKCδ: Cellular Uptake, Molecular Docking, and Binding Affinity

**Figure 6:**
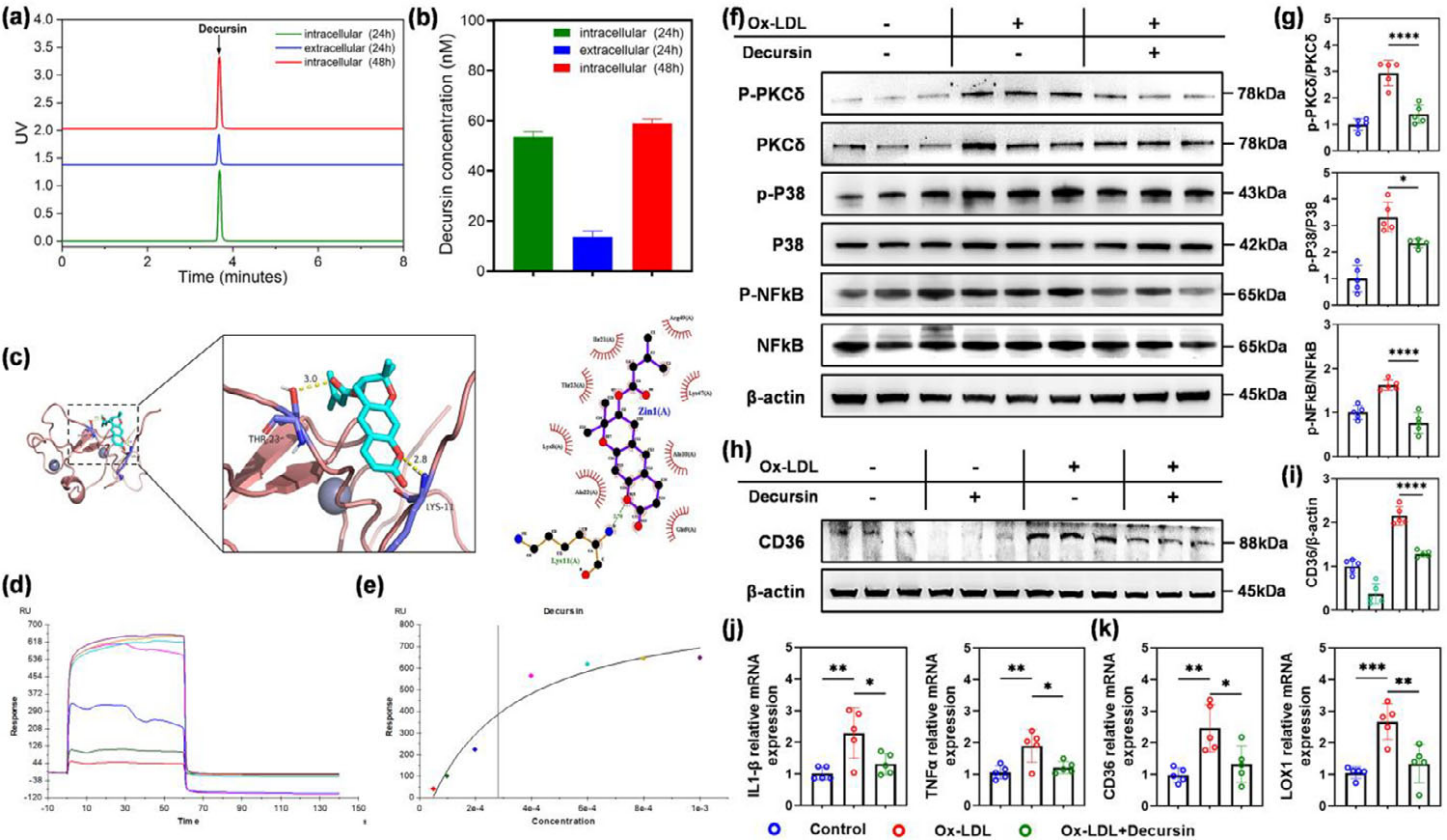
Decursin Interacts with PKCδ and Regulates Inflammatory Responses and Lipid Accumulation in Macrophages a,b) Quantification of the intracellular and extracellular decursin concentrations at 24 and 48 h using high‐performance liquid chromatography (HPLC). c) Molecular docking simulation of decursin complexed with PKCδ. d) Response value reflecting the interaction between decursin and PKCδ. e) Affinity fitting curves corresponding to the reported gradient concentrations. f,g) Representative immunoblots of phosphorylated (p‐) and total PKCδ, p38, NF‐κB, and CD36 in RAW264.7 cells treated with Ox‐LDL. h,i) Densitometric quantification of the immunoblots shown in (f, g). β‐actin was used as a loading control (*n* = 5). j,k) Relative mRNA expression levels of *IL‐1β*, *TNF‐α*, *CD36*, and *LOX1* in Ox‐LDL‐treated RAW264.7 cells were detected via qRT‒PCR (*n* = 5). The data are expressed as the means ± s.d.s. NS indicates not significant. ^*^ *p* < 0.05, ^**^ *p* < 0.01, ^***^ *p* < 0.001, and ^****^ *p* < 0.0001.

To elucidate the pharmacokinetic behavior of decursin at the cellular level and determine its primary site of action, we conducted comprehensive localization studies in RAW264.7 macrophages under atherosclerotic conditions. The cells were treated with oxidized low‐density lipoprotein to mimic atherosclerotic environments, followed by the administration of decursin. At predetermined time points (24 and 48 h), we meticulously separated and collected both the cell culture supernatants and the intracellular fractions following standardized protocols, including trypsinization, membrane disruption, and differential centrifugation, to ensure the complete extraction of decursin from the subcellular compartments. The concentrations of decursin in both the cell culture supernatant and the cell lysates were quantified using HPLC (**Figure** **6**a; Figure S5, Supporting Information). After 24 h, the decursin levels in the cell lysates were significantly greater than those in the supernatant, and this disparity became even more pronounced at 48 h, with decursin becoming nearly undetectable in the supernatant (Figure 6b). These findings indicate that decursin predominantly localizes within cells over time, suggesting that its therapeutic effects are likely mediated intracellularly.

On the basis of the intracellular accumulation of decursin, we explored its potential molecular interactions with intracellular targets. Molecular docking simulations using AutoDock and visualizations with PyMol revealed that decursin interacts with protein kinase C delta by forming hydrogen bonds with the threonine residue at position 23 (Thr‐23) and the lysine residue at position 11 (Lys‐11), whereas the active site of PKCδ remains relatively stable (Figure 6c). These findings suggest a specific binding mode that could influence PKCδ function without altering the conformation of its active site. To further validate the interaction between decursin and PKCδ, we performed surface plasmon resonance (SPR) experiments. The SPR analyses revealed “rapid association/rapid dissociation” kinetics (Figure 6d), indicating direct and transient binding interactions. The equilibrium dissociation constant (KD) for the decursin‐PKCδ interaction was determined to be 2.81 × 10^−4^ M (Figure 6e; Figure S6, Supporting Information), confirming the moderate affinity between decursin and PKCδ. Collectively, these integrated pharmacokinetic and molecular interaction studies provide compelling evidence that decursin accumulates intracellularly in macrophages and forms specific, direct interactions with PKCδ. The observed binding kinetics and molecular interaction profile strongly suggest that decursin functions as an allosteric modulator of PKCδ signaling pathways, which are critically involved in macrophage inflammatory responses, lipid metabolism, and foam cell formation in the context of atherosclerosis progression. These findings not only elucidate the mechanism of action of decursin but also identify PKCδ as a potential therapeutic target for atherosclerosis intervention.

### 2.8 Decursin Inhibits PKCδ Activation to Regulate Inflammatory Responses and the Lipid Accumulation Pathway

The present study highlights an interaction between decursin and PKCδ. To explore this interaction and its downstream effects, we evaluated the phosphorylation status of key signaling proteins. Ox‐LDL stimulation significantly increased the phosphorylation of PKCδ, p38 MAPK, and NF‐κB in RAW264.7 cells, indicating proinflammatory activation. Decursin treatment notably reduced the Ox‐LDL‐induced hyperphosphorylation of PKCδ, p38 MAPK, and NF‐κB (Figure 6f,g). Additionally, decursin downregulated the protein expression of CD36, a scavenger receptor associated with foam cell formation and atherogenesis (Figure 6h,i). Consistent with these results, decursin also inhibited the mRNA expression of the proinflammatory cytokines IL‐1β and TNFα (Figure 6j), as well as the lipid accumulation‐related markers CD36 and LOX‐1 (Figure 6k). Collectively, these findings suggest that decursin penetrates macrophages, binds to PKCδ, and modulates key signaling pathways involved in inflammation and lipid metabolism. This mechanistic insight positions decursin as a promising therapeutic agent for reducing macrophage‐mediated inflammatory responses and lipid accumulation, offering potential benefits in managing atherosclerosis and related cardiovascular diseases.

## 3 Conclusion

In this study, we demonstrated that decursin, a plant‐derived monomer, has therapeutic efficacy against atherosclerotic plaque formation by inhibiting lipid accumulation and modulating inflammatory responses while showing minimal cytotoxicity. Mechanistically, decursin directly binds to PKCδ, antagonizing the phosphorylation of PKCδ induced by Ox‐LDL. This interaction effectively suppresses the activation of downstream PKCδ pathways, reducing inflammatory responses and the expression of proteins linked to lipid accumulation. However, the therapeutic potential of decursin is limited by its short lifespan, which limits its ability to sustain plaque inhibition. To address this limitation, we developed the nanovesicle ALD@EM, which increases the stability of decursin and incorporates macrophage‐targeting properties, aligning with the systemic processes involved in atherosclerotic plaque development. Our findings indicate that ALD@EM significantly increases the bioavailability and targeted delivery of decursin, thereby amplifying its antiatherosclerotic effects. Consequently, ALD@EM represents a promising nanocarrier system for increasing the therapeutic efficacy of antiatherosclerotic agents such as decursin, offering potential advancements in the management and treatment of atherosclerosis.

## 4 Experimental Section

### Materials

The following materials were used in this study: decursin (SML0786, Sigma‒Aldrich); Mem‐PERTM Plus Membrane Protein Extraction Kit (89 842, Thermo Fisher); Oil Red O Staining Kit (C0157S, Beyotime), Nile Red (72 485, Sigma‒Aldrich), Coomassie Brilliant Blue Staining (P0017, Beyotime); dimethyl sulfoxide (276 855, Sigma‒Aldrich), DSPE‐PEG‐NHS (HY‐W441014, MedChemExpress), low density lipoprotein (L3486, Thermo Fisher); Enhanced Cell Counting Kit‐8 (C0041, Beyotime); DiD (C1039, Beyotime); Calcein/PI Live/Dead Cell Viability/Cytotoxicity Assay Kit (C2015 M, Beyotime); and antibodies against ICAM‐1 (ab171123, Abcam), VCAM‐1 (MAB6432, RD Systems), PKCδ (9616T, CST), phospho‐PKCδ (9374S, CST), p38 MAPK (8690S, CST), phospho‐p38 MAPK (4511S, CST), NF‐κB (8242S, CST), phospho‐NF‐κB (3033S, CST), β‐actin (4970S, CST), and CD36 (14347S, CST).

### MUVEC Membrane Protein Extraction

Membrane proteins were extracted via a Mem‐PER Plus kit (89 842, Thermo Scientific). The MUVECs were washed, resuspended in buffer, and centrifuged. The cells were permeabilized and centrifuged at 16 000 × g. The pellet was resuspended in a solubilization buffer and incubated at 4 °C with gentle agitation. The membrane protein‐containing supernatant was collected after the final centrifugation and stored at −80 °C until use.

### Preparation of ALD@EM

ALD@EM was prepared by a thin film dispersion‐extrusion process according to previous methods.^[^ ^32^ ^]^ Crude liposomes containing decursin were prepared via the classic thin‐film dispersion method, and then, a coextrusion method was used to coassemble the apoptotic endothelial membrane and antibody components to generate biomimetic ALD@EMs.

Prior to the coextrusion step, antibody‐conjugated PEGylated phospholipids, including DSPE‐PEG‐ICAM1‐Ab and DSPE‐PEG‐VCAM1‐Ab, were synthesized according to previous methods.^[^ ^33^ ^]^ Briefly, DSPE‐PEG‐NHS was dissolved in dimethyl sulfoxide (DMSO) to prepare a stock solution at a concentration of 10 mg mL^−1^. A small amount of DSPE‐PEG‐NHS stock solution was added to the antibodies (ICAM1‐Ab or VCAM1‐Ab) dissolved in PBS (pH 7.4) at a final molar ratio of 20:1. The mixture was gently stirred at room temperature for 2 h. Upon completion of the reaction, glycine was added to a final concentration of 50 mM to quench the remaining active NHS esters, followed by incubation at room temperature for 10 min. The reaction mixture was then purified using an ultrafiltration tube (MWCO 10 kD) to remove unreacted DSPE‐PEG‐NHS and other small molecules. The final antibody‐DSPE‐PEG conjugate was reconstituted in PBS and stored at 4 °C.

After that, liposomes containing decursin were prepared via the classic thin‐film dispersion method. Briefly, the lipid components, including lecithin, cholesterol, and decursin (65:30:5, mol mol^−1^), were dissolved in a chloroform/methanol mixture (3:1, v/v) in a round‐bottom flask. The organic solvents were subsequently removed under reduced pressure using a rotary evaporator to generate a thin, uniform lipid film on the inner wall of the flask. The lipid film was then hydrated with PBS (pH 7.4) at 37 °C for 30 min to produce a crude liposome suspension. Subsequently, the crude liposome suspension was mixed with the apoptotic endothelial membrane, LDL, DSPE‐PEG‐ICAM1‐Ab, and DSPE‐PEG‐VCAM1‐Ab in PBS buffer. The mixture was passed through a high‐pressure extrusion system equipped with polycarbonate membranes. The mixture was extruded through a 450 nm membrane 5 times. The final ALD@EM was purified to remove unencapsulated biomolecules using ultracentrifugation.

### Preparation of Liposomes

The lipid components (lecithin:cholesterol = 65:30, mol mol^−1^) were dissolved in chloroform/methanol (3:1, v/v) in a round‐bottom flask. The organic solvents were removed using a rotary evaporator (Buchi R‐210, Switzerland) at 37 °C under reduced pressure (first at 100 mbar for 15 min and then at 50 mbar for 30 min) to form a thin, uniform lipid film. The flask was placed in a vacuum desiccator overnight to remove residual solvent. The lipid film was hydrated with PBS (pH 7.4) at 37 °C for 30 min with gentle rotation (120 rpm) to yield a crude liposome suspension at a final lipid concentration of 10 mg mL^−1^. The suspension was then subjected to probe sonication (Sonics Vibra‐Cell, USA) for 5 min (amplitude: 40%, pulse on/off: 5 s/5 s) in an ice bath to reduce the particle size before extrusion.^[^ ^34^ ^]^

### Preparation of Apoptotic EM Nanovesicles

Apoptotic cells were collected by centrifugation (500 × g, 5 min), washed three times with cold PBS, and resuspended in homogenization buffer (10 mM Tris‐HCl, 1 mM EDTA, 0.2 mM PMSF, pH 7.4). The cell suspension was homogenized using a Dounce homogenizer (20 strokes) and centrifuged at 700 × g for 10 min at 4 °C to remove the nuclei and unbroken cells.^[^ ^35^ ^]^ The supernatant was collected and ultracentrifuged at 100000 × g for 1 h at 4 °C. The membrane pellet was resuspended in PBS, and the protein content was quantified using the BCA assay. The purified membrane was then extruded through 200 nm polycarbonate membranes (21 passes) using an Avanti Mini‐Extruder (Avanti Polar Lipids, USA) to form EM nanovesicles.^[^ ^36^ ^]^ The resulting EM nanovesicles were stored at 4 °C and used within one week.

### Characterization of ALD@EM

Liposome characterization included particle size analysis and zeta potential measurements performed on a ZS90 system (Malvern Instruments, UK). The ultrastructural features were examined by TEM utilizing a Spirit Twin microscope (FEI, USA, Tecnai G2 model).

### Coomassie Brilliant Blue Staining

The gel was submerged in deionized water (50 mL) and microwaved for 3 min. After the liquid was removed, the gel was stained with Coomassie Brilliant Blue rapid staining solution (20 mL, 10–30 min). The gel was subsequently destained in deionized water (100 mL) with continuous agitation, and the solution was replaced every 5–15 min until the optimal contrast was achieved (30–120 min).

### Construction of the Atherosclerosis Model in ApoE‐/‐ Mice

Male ApoE‐/‐ mice (8‐week‐old, 20–22 g, Cyagen Biosciences, Suzhou, China) were housed in specific‐pathogen‐free facilities (temperature: 20 ± 2 °C, humidity: 55 ± 5%, 12‐h light/dark cycle) with free access to standard chow and water during acclimatization. The mice were housed 5 per cage in ventilated cages with corn cob bedding. Atherosclerosis was induced by feeding mice a well‐characterized high‐fat diet (1.25% Added Cholesterol and 0.5% Sodium Cholate; D12109C, Research Diets, New Brunswick, NJ, USA) for 14 weeks. Food consumption (daily) and body weight (weekly) were monitored throughout the study. For confirmation of atherosclerosis progression, three randomly selected mice were sacrificed at week 8, and aortic tissues were collected for histological analysis of early plaque formation using Oil Red O and H&E staining. All procedures were approved by the Animal Welfare and Ethics Committee of Yunnan University (YNU20220231) and conducted in accordance with NIH guidelines (NIH Publication 85‐23, Rev. 1985).

### Induction of Foam Cell Formation

RAW 264.7 macrophages (ATCC TIB‐71) were maintained in DMEM (Gibco 11 965 092) supplemented with 10% FBS and 1% penicillin‒streptomycin at 37 °C with 5% CO₂. The cells were seeded (2 × 10⁵ cells/well) in 6‐well plates and grown to 70–80% confluence. After 6 h of serum starvation, the cells were treated with Ox‐LDL (50 µg mL^−1^; YB‐002, Yuan Ye) for 24 h to induce foam cell formation, which was confirmed as previously described.^[^ ^37^ ^]^

### Oil Red O Staining of Aortas

Following euthanasia under isoflurane anesthesia, aortas were excised from the root to the iliac bifurcation and cleaned of adventitial tissue. The samples were fixed in 10% neutral‐buffered formalin (4 °C, overnight) and rinsed with PBS. For Oil Red O staining, aortas were primed in 60% isopropanol (5 min), stained with Oil Red O solution (C0157S, Beyotime; 1 h, room temperature) in the dark, and differentiated in 60% isopropanol (5 min). After longitudinal dissection, en face images were captured under a stereomicroscope. Atherosclerotic lesions were quantified using ImageJ_V1.52a and expressed as a percentage of the total aortic surface area.

### Nile Red Staining

Nile Red stock solution (1 mg mL^−1^ in acetone; Sigma, 72 485) was diluted to 1 µg mL^−1^ in PBS. Ox‐LDL‐treated cells were fixed with 4% paraformaldehyde (15 min, room temperature) and stained with Nile red solution (10 min, room temperature) in the dark. Lipid droplets were visualized using confocal microscopy (LSM 880, Zeiss) with excitation at 488 nm, and emission was measured at 520–560 nm.

### Macrophage Targeting Assay In Vitro

For evaluation of the macrophage‐targeting efficacy, Ox‐LDL‐stimulated RAW264.7 cells (24 h) were incubated with DiD‐labeled EM or ALD@EM (1 h). After being washed with PBS, the cells were stained with calcein‐AM (20 min). Fluorescence images were captured using an Olympus BX61TRF microscope (DiD: Ex 640 nm/Em 680 nm). Nanovesicle uptake was quantified by ImageJ (version 1.2a) analysis.

### Atherosclerotic Plaque Targeting Assessment In Vivo

Atherosclerotic mice were randomly assigned to receive PBS, DiR‐labeled EM, or ALD@EM via intravenous injection. The aortic distribution of the nanocarriers was monitored via an IVIS Lumina III (PerkinElmer; Ex 745 nm/Em 800 nm) at 12, 24, and 48 h post‐injection. The time‐dependent DiR fluorescence intensity was quantified.

### Measurement of Inflammatory Cytokines

Inflammatory cytokines were quantified via a RayPlex Mouse Inflammation Array (FAM‐INF‐1‐96; RayBiotech) according to the manufacturer's protocol.

### Histological Evaluation

After 14 weeks of HFD feeding, ApoE‐/‐ mice were sacrificed. The hearts, lungs, livers, spleens, and kidneys were harvested, perfused with PBS followed by 4% paraformaldehyde, and subsequently embedded in paraffin. Tissue sections were prepared and stained using hematoxylin and eosin (H&E) and Masson's trichrome staining protocols. Microscopy images were acquired using a Nikon H500S imaging system and analyzed with K‐viewer software.^[^ ^38^ ^]^

### Echocardiography

Left ventricular (LV) function was evaluated using the Vevo 2100 imaging system (VisualSonics), as described previously.^[^ ^39^ ^]^ Briefly, the mice were anesthetized with 1.5% isoflurane via inhalation and placed in the supine position on a heated surgical stage with the limbs secured. The ultrasound probe was placed perpendicular to the chest for imaging. Echocardiographic data and M‐mode images were collected and analyzed at 14 weeks after HFD feeding in ApoE‐/‐ mice. Key echocardiographic parameters, including ejection fraction (EF), fractional shortening (FS), stroke volume (SV), cardiac output (CO), LV end‐diastolic diameter (LVEDD), and LV end‐systolic diameter (LVESD), were automatically calculated using the “LV TRACE” mode, which tracks endocardial borders.

### HPLC System

As previously described,^[^ ^40^ ^]^ a Beckman System Gold HPLC system consisting of a 126 solvent module, a 507e autosampler, and a diode array detector module 168 was used. Chromatographic separation was performed at room temperature on a 5 µm Clipeus C18 column (250 mm × 4.6 mm, Higgins Analytical) (Waters, WAT054275). The mobile phase comprised water (solvent A) and 95% acetonitrile (solvent B), with a linear gradient from 30% B to 60% B over 8 min at a flow rate of 0.5 mL min^−1^. Detection was carried out at a wavelength of 330 nm, with an injection volume of 10 µL.

### Verification of the Results of the Molecular Docking Analysis and Surface Plasmon Resonance (SPR) Assay

The protein structure was retrieved from the PDB database, and the decursin structure was retrieved from PubChem. Molecular docking was performed using AutoDock Vina. For SPR analysis (Biacore T200, GE Healthcare), protein samples (30 µg mL^−1^ in 10 mM sodium acetate, pH 4.5) were immobilized on a CM5 chip. Decursin (1000 µM) was injected at 10 µL min^−1^ with 120 s of association and 180 s of dissociation in PBST buffer (25 °C). The data were analyzed using Biacore T200 evaluation software.

### Western Blotting

Protein lysates (30 µg) were separated by 10% SDS‒PAGE and transferred to PVDF membranes (IPFL00010, Millipore). After blocking, the membranes were probed with primary antibodies (1:1000; phospho‐PKCδ, PKCδ, phospho‐p38 MAPK, p38 MAPK, phospho‐NF‐κB p65, NF‐κB p65, and β‐actin) overnight at 4 °C, followed by incubation with an HRP‐conjugated secondary antibody (1:5000, 2 h, room temperature). Proteins were visualized using HRP substrate (WBKLS0500, Millipore), with β‐actin serving as a loading control.^[^ ^41^ ^]^

### RNA Extraction and Quantitative Real‐Time PCR

Total RNA was extracted using TRIzol (15596026CN; Invitrogen) and reverse transcribed using PrimeScript RT Master Mix (RR036A; TaKaRa). qRT‒PCR was performed with TB Green Premix Ex Taq (RR820A, TaKaRa) on a QuantStudio 6 Flex system. Gene expression was normalized to that of 36b4 using the ∆∆CT method.^[^ ^42^ ^]^

### Statistical Analysis

Unless otherwise stated, average data are shown as the mean and SEM. Dunnett's multiple comparison test was used for one‐way ANOVA to compare > 2 groups. Two‐way ANOVA and Bonferroni post hoc correction were used to compare groups with > 1 factor to test the interaction between factors. Repeated‐measures ANOVA with Dunnett's multiple comparison tests was used to study the changes in the average score at ≥3 time points. Student's *t*‐test was used to compare two normally distributed values. *p* <0.05 was considered statistically significant. Statistical analysis was performed using GraphPad Prism 7.0 (GraphPad Prism software) and SPSS 14.0 (IBM Corp) for Windows.

## Conflict of Interest

The authors declare no conflict of interest.

## Author Contributions

H.C., Y.Z., and M.A. contributed equally to this work. Y.L., K.Y., W.C., and Y.D. conceived the idea, designed the study, analyzed and interpreted the data, and wrote the paper. H.C., Y.Y., M.A., Y.Z., and S.S. performed the experiments. Y.W., J.C., L.Z., and Q.Z. helped to perform the in vivo experiments. F.H. and Y.P.L. helped to collect tissues and perform echocardiography tests. Y.L., K.Y., W.C., and Y.D. supervised the entire project. All the authors discussed the results and commented on the paper.

## Supporting information



Supporting Information

## Data Availability

Research data are not shared.
