# Supplementary material for: Decursin‐Loaded Nanovesicles Target Macrophages Driven by the Pathological Process of Atherosclerosis
Source: Adv Sci (Weinh). 2025 Apr 26;12(22):2417489. doi: 10.1002/advs.202417489 (PMC12165033; doi:10.1002/advs.202417489)
Supplement: Supplementary file 1 — Supporting Information [file ADVS-12-2417489-s001.docx]

**Supporting Information**

**Decursin-Loaded nanovesicles targeted macrophages Driven by the Pathological Process of Atherosclerosis**

Hui Chen^1, 3*^, Yifeng Zhang^4*^, Mirenuer Aikebaier^4*^, Yawei Du^2^, Yan Liu^3^, Qing Zha^3^, Lan Zheng^5^, Shuyao Shan^4^, Yanping Wang^3^, Jiawei Chen^3^, Yiping Li^1^, Ke Yang^4#^, Ying Yang^1#^, Wenguo Cui^2#^

1. Department of Endocrinology, the Affiliated Hospital of Yunnan University, Kunming, Yunnan, China.
2. Department of Orthopaedics, Shanghai Key Laboratory for Prevention and Treatment of Bone and Joint Diseases, Shanghai Institute of Traumatology and Orthopaedics, Ruijin Hospital, Shanghai Jiao Tong University School of Medicine, Shanghai, China.
3. Department of Cardiology, Shanghai Ninth People's Hospital, Shanghai Jiao Tong University School of Medicine, Shanghai, China.
4. Department of Cardiovascular Medicine, Ruijin Hospital, Shanghai Jiao Tong University School of Medicine, Shanghai, China.
5. Department of Traditional Chinese Medicine, Ruijin Hospital, Shanghai Jiao Tong University School of Medicine, Shanghai, China.

* Contributed equally to this study; #co-corresponding author

**Correspondence author:**

Wenguo Cui, MD, Department of Orthopaedics, Shanghai Key Laboratory for Prevention and Treatment of Bone and Joint Diseases, Shanghai Institute of Traumatology and Orthopaedics, Ruijin Hospital, Shanghai Jiao Tong University School of Medicine, 197 Ruijin 2nd Road, Shanghai, 200025, China. E-mail: [wgcui@sjtu.edu.cn](mailto:wgcui@sjtu.edu.cn)

**Co-corresponding author:**

Ying Yang, MD, Department of Endocrinology, the Affiliated Hospital of Yunnan University, Qingnian Road 176, Kunming, Yunnan, 650091, China. E-mail: yangying2072@126.com

Ke Yang, PhD, Department of Cardiovascular Medicine, Ruijin Hospital, Shanghai Jiao Tong University School of Medicine, No. 197 Ruijin Second Rd, Shanghai 200025, China. E-mail: [ykkykkk@sjtu.edu.cn](mailto:ykkykkk@sjtu.edu.cn)


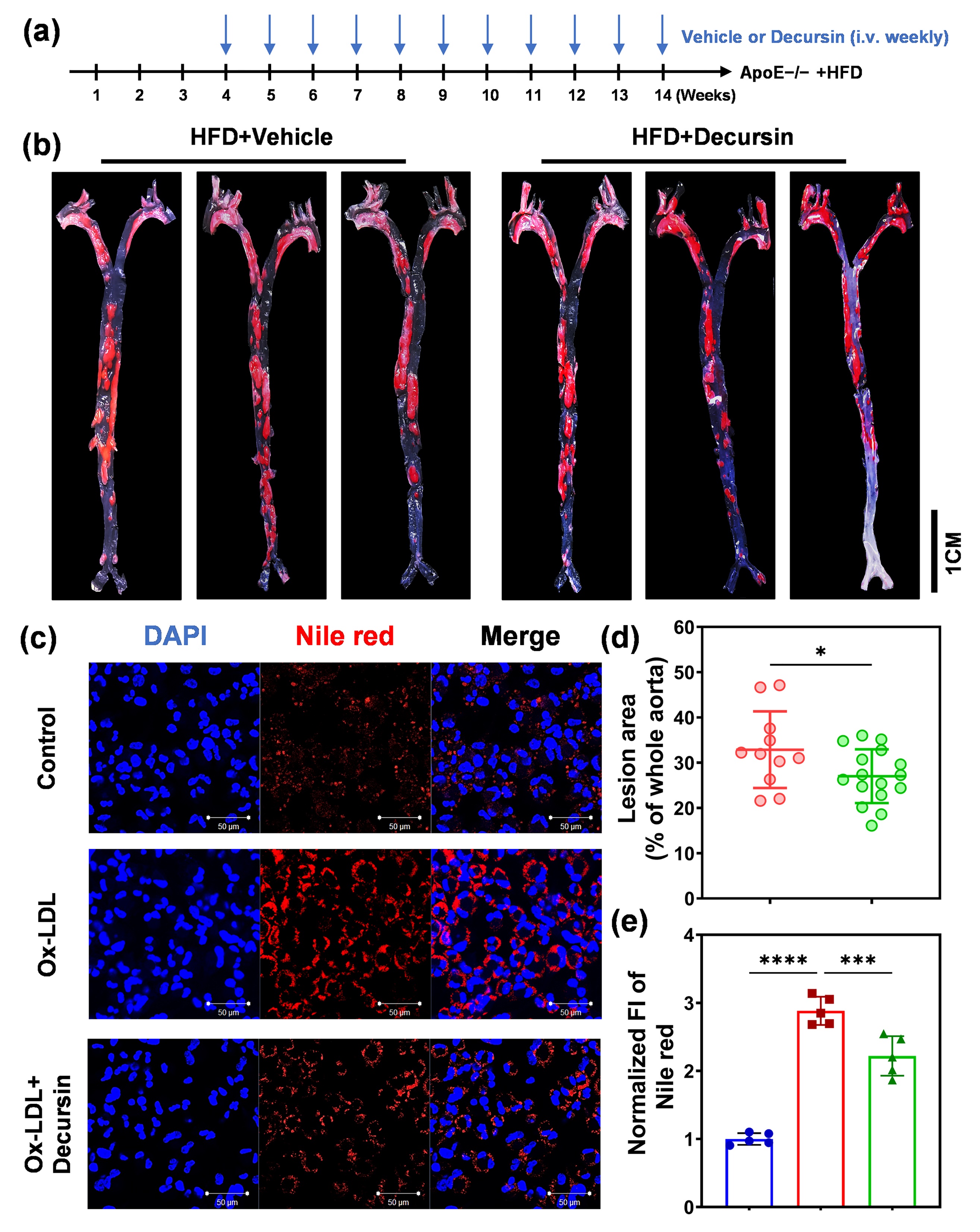


**Figure S1.** Therapeutic Efficacy of Decursin *In Vivo* and *In Vitro*. (a) Schematic representation of the experimental timeline for decursin treatment in ApoE-/- mice fed a high-fat diet (HFD). (b) Representative images of Oil Red O (ORO) staining of aortas from vehicle and decursin treated mice at 14 weeks after HFD. (c) Representative confocal microscopy images of Nile Red staining in RAW264.7 cells treated with vehicle, Ox-LDL, or Ox-LDL + decursin for 24 hours. (d) Quantification of the ORO-positive lesion area relative to the whole aorta (n = 10). (e) Quantification of Nile Red fluorescence intensity in RAW264.7 cells, representing intracellular lipid accumulation (n = 5). The data are expressed as mean ± s.d. NS indicates not significant. *P< 0.05, ***P< 0.001, and ****P< 0.0001.


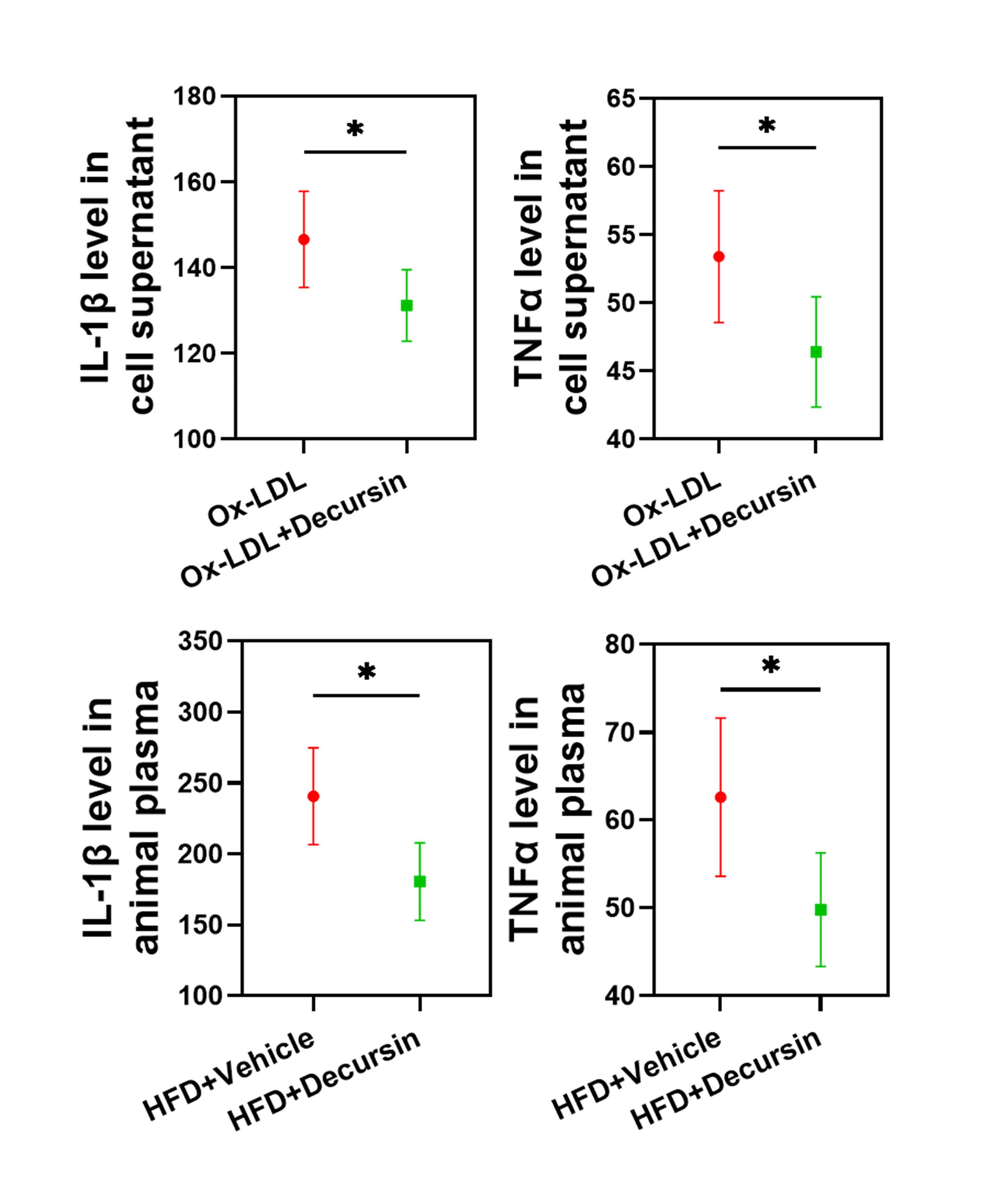


**Figure S2.** Decursin Treatment Reduces IL-1β and TNF-α Levels. Quantitative analysis of IL-1β and TNF-α levels in the decursin treatment group compared to the vehicle control group. The results indicate a significant decrease in the levels of these pro-inflammatory cytokines following decursin treatment (n = 5). The data are expressed as mean ± s.d. NS indicates not significant. *P< 0.05.


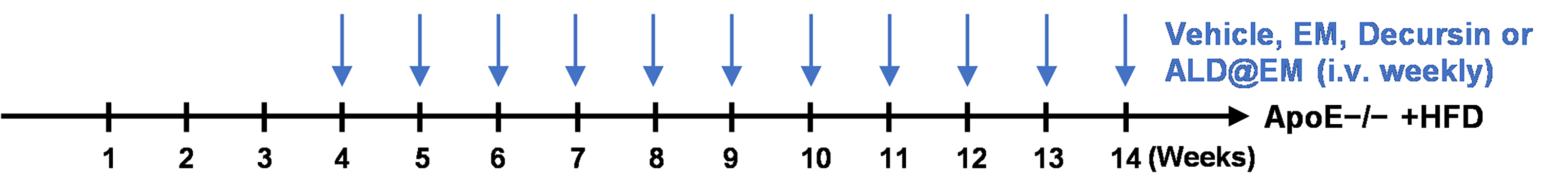


**Figure S3.**Schematic representation of the experimental protocol for evaluating the long-term efficacy of ALD@EM *in vivo*.


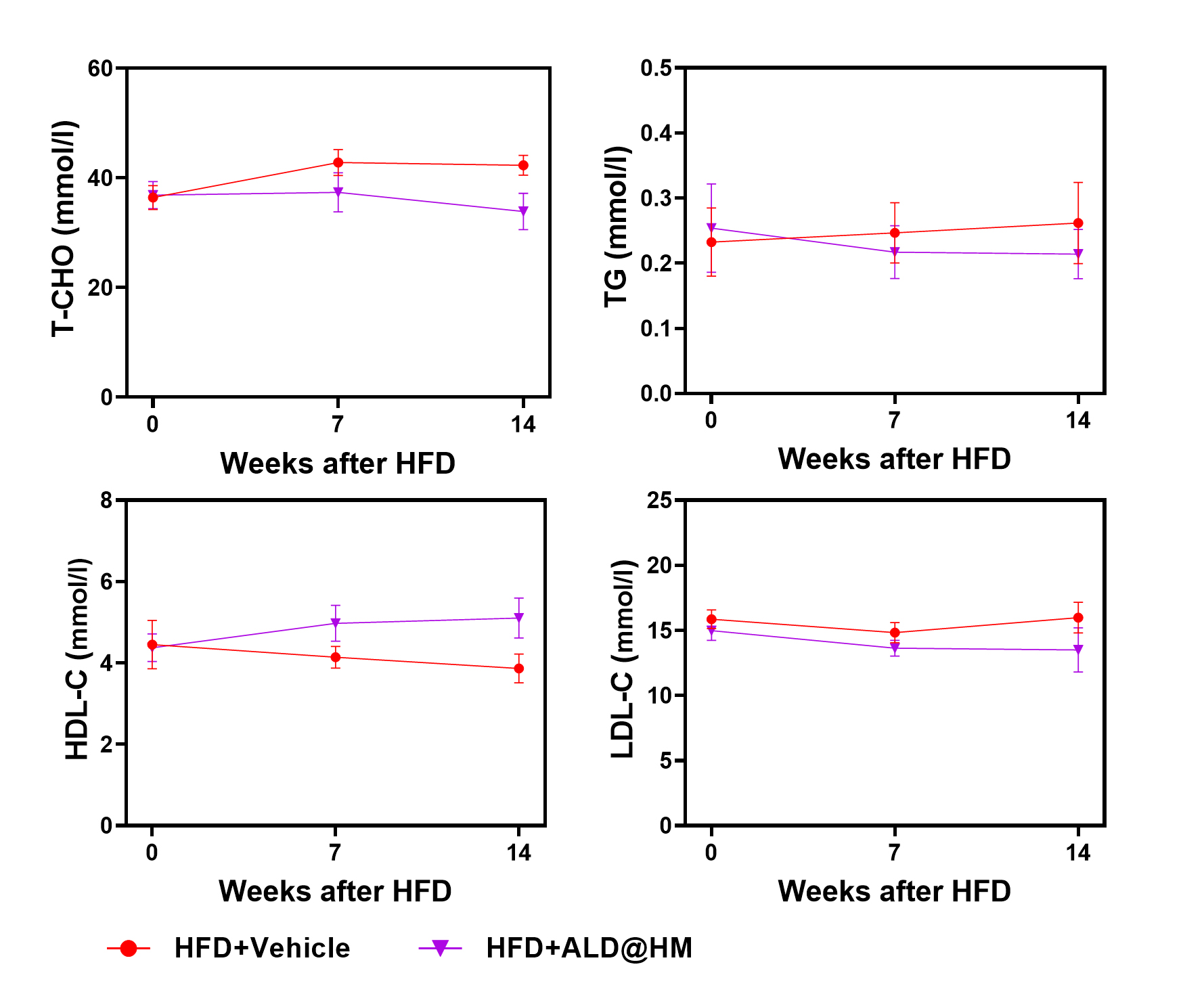


**Figure S4.** Quantitative Analysis of Serum Lipid Levels in ApoE-/- Mice. Quantitative analysis of Serum levels of total cholesterol (T-CHO), triglycerides (TG), low-density lipoprotein cholesterol (LDL-C), and high-density lipoprotein cholesterol (HDL-C) in ApoE-/- mice at 14 weeks after HFD. Mice were treated with ALD@EM, or vehicle.


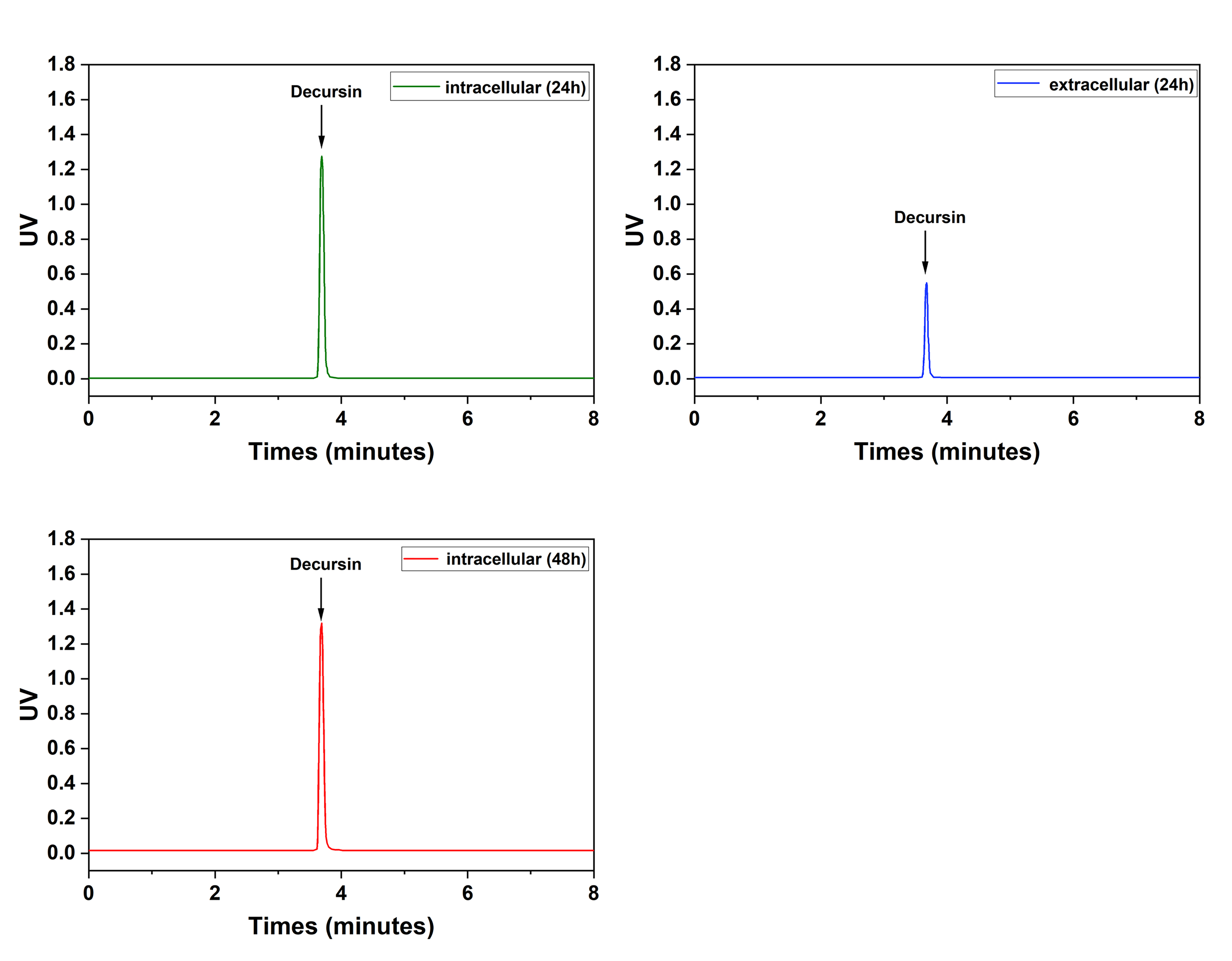


**Figure S5.** Cellular Uptake of Decursin by RAW264.7 Macrophages. Representative HPLC chromatograms showing decursin levels in cell supernatant and cell lysates of Ox-LDL-stimulated RAW264.7 macrophages after incubation with decursin for 24 and 48 hours.


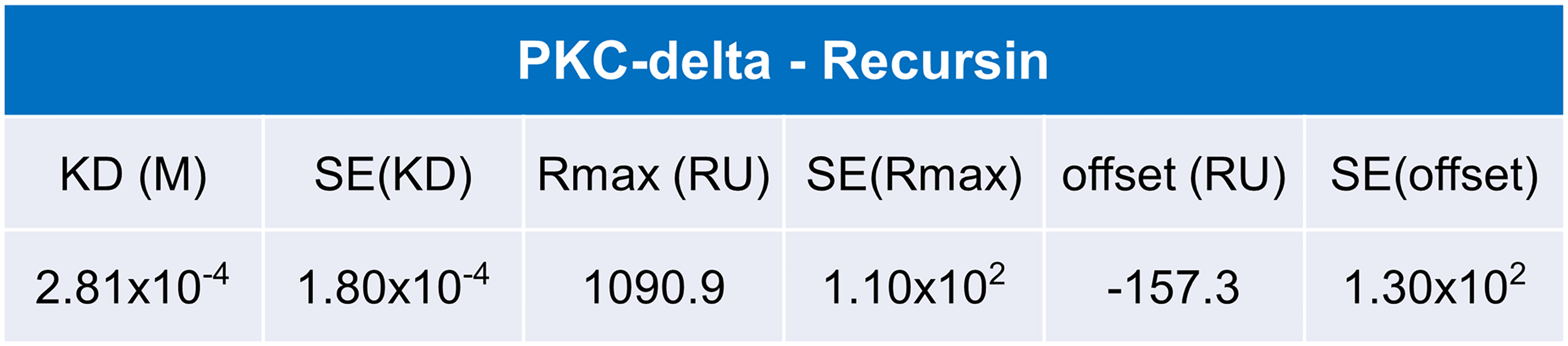


**Figure S6.** Determination of the Binding Affinity of Decursin to PKCδ. Sensorgrams showing the interaction between decursin and PKCδ obtained by surface plasmon resonance. The equilibrium dissociation constant was determined to be 2.81 × 10^-4^ M.

**Table S1.** Quantitative real-time PCR primers

| **Gene** | **Direction** | **Sequence 5'→3'** |
| --- | --- | --- |
| IL1-β | Forward | GCAACTGTTCCTGAACTCAACT |
|  | Reverse | ATCTTTTGGGGTCCGTCAACT |
| TNF-α | Forward | GACGTGGAACTGGCAGAAGAG |
|  | Reverse | TTGGTGGTTTGTGAGTGTGAG |
| CD36 | Forward | ATGGGCTGTGATCGGAACTG |
|  | Reverse | GTCTTCCCAATAAGCATGTCTCC |
| LOX1 | Forward | CAAGATGAAGCCTGCGAATGA |
|  | Reverse | ACCTGGCGTAATTGTGTCCAC |
